# From Spatial Epigenomes to Clinical Diagnostics: Integrative Methylomics Across Scales and Modalities

**DOI:** 10.3390/ijms27104377

**Published:** 2026-05-14

**Authors:** Aiman Kinzhebay, Aina Zhanymbetova, Ainur Yerkos, Zhibek Zhetpisbay, Rustem Imanbek, Amankeldi A. Salybekov

**Affiliations:** 1 Regenerative Medicine Division, Cell and Gene Therapy Department, Qazaq Institute of Innovative Medicine, Astana 010000, Kazakhstan; a.kinzhebay@qaziim.kz (A.K.); uaisovaaina96@gmail.com (A.Z.); 2 Department of Oncology, Astana Medical University, Astana 010000, Kazakhstan; 3 Department of Computer Science, Al-Farabi Kazakh National University, Al-Farabi Avenue, Almaty 050040, Kazakhstan; yerkosova@gmail.com (A.Y.); zhetpisbay_zhibek1@live.kaznu.kz (Z.Z.); imanbek_rustem4@live.kaznu.kz (R.I.)

**Keywords:** DNA methylation, epigenomics, spatial methylomics, cell-free DNA (cfDNA), long-read sequencing, epigenetic biomarkers, cancer epigenetics

## Abstract

Methylomics has emerged as a central framework for understanding gene regulation in development and disease, yet the rapid expansion of profiling technologies, computational integration methods, and clinical applications has outpaced comprehensive synthesis. This review addresses that gap by systematically examining current advances across the full methylomics pipeline, from data generation to clinical translation. We draw on evidence from large-scale consortium datasets and benchmarking studies of multi-omics integration methods including MOFA, DIABLO, and deep learning architectures, single-cell and spatial methylomic technologies, long-read sequencing platforms (Oxford Nanopore, PacBio HiFi), and cell-free DNA (cfDNA) liquid biopsy approaches. The review further surveys methylation dysregulation across major disease domains, including cancer, cardiovascular disease, neurological disorders, and autoimmune conditions. Integrating methylomic data with transcriptomic and chromatin accessibility layers, particularly in spatial and single-cell contexts, substantially improves the resolution of disease-associated regulatory mechanisms. cfDNA methylation profiling emerges as a cross-disease, non-invasive monitoring platform with broad diagnostic potential, supported by machine learning-based deconvolution. We conclude that while technological barriers are diminishing, standardization of analytical workflows, population diversity in reference datasets, and regulatory alignment remain the principal challenges for translating methylomics advances into broadly accessible precision medicine.

## 1. Introduction

DNA methylation—the covalent addition of a methyl group to the C5 position of cytosine predominantly within CpG dinucleotides—represents one of the most mechanistically characterized epigenetic modifications in mammalian biology. Catalyzed by the DNA methyltransferase (DNMT) family, with *DNMT3A* and *DNMT3B* executing de novo methylation and *DNMT1* maintaining patterns during replication [1], this modification operates through two principal regulatory mechanisms. At promoter CpG islands (CGIs) genomic regions of ≥200 bp, >50% GC content, and elevated CpG density hypermethylation silences transcription by occluding transcription factor binding sites and recruiting methyl-CpG-binding domain (MBD) proteins that scaffold histone deacetylase repressor complexes [2], whereas gene body methylation paradoxically correlates positively with active transcription by suppressing spurious intragenic initiation [3]. Reversibility through the ten-eleven translocation (TET) dioxygenase family, which sequentially oxidizes 5-methylcytosine (5mC) to 5-hydroxymethylcytosine (5hmC) and beyond, ultimately restoring unmodified cytosine via base-excision repair [4], endows the methylome with dynamic responsiveness to developmental cues and environmental stimuli, positioning it as both a heritable cell-identity barcode and a sensitive readout of physiological and pathological state.

Over the past four decades, the methodological landscape for interrogating DNA methylation has evolved from low-throughput, locus-specific assays toward genome-wide, single-cell, and spatially resolved approaches [5,6]. Early tools such as restriction landmark genomic scanning (RLGS, ~1991) and methylation-specific PCR provided binary readouts at predefined loci, sufficient for single-gene biomarker validation but incompatible with systematic discovery [6,7]. The introduction of sodium bisulfite conversion established the biochemical foundation for modern sequencing-based assays by enabling cytosine-to-uracil conversion at unmethylated cytosines, rendering 5mC distinguishable at single-base resolution and paving the way for successive generations of high-throughput profiling methods [6,7].

Array-based platforms, including the Illumina 450K BeadChip (~485,000 CpG sites), EPIC v1.0 (~850,000 sites), and the expanded EPIC v2.0 (~935,000 sites, 2022), democratized population-scale epigenome-wide association studies and anchored large consortium efforts such as The Cancer Genome Atlas and the Roadmap Epigenomics Consortium, generating invaluable reference methylomes and enabling biomarker discovery across thousands of samples [8,9,10]. Whole-genome bisulfite sequencing and its enzymatic counterpart EM-seq subsequently provided single-CpG resolution across the entire mappable genome, capturing the full repertoire of methylation variation, including non-CpG and hydroxymethylcytosine contexts inaccessible to array platforms Evolution of genome-wide methylation profiling technologies [5]. Despite their remarkable productivity, however, both array and WGBS approaches applied to tissue homogenates are fundamentally bulk measurements that irretrievably conflate signals from heterogeneous cell mixtures, rendering the cellular origins of disease-associated differentially methylated regions ambiguous and precluding analysis of the spatial logic that governs epigenomic organization within intact tissues scMET: Bayesian modeling of DNA methylation heterogeneity at single-cell resolution [11] and s tatistical and integrative system-level analysis of DNA methylation data [10].

The advent of single-cell DNA methylation sequencing, including snmC-seq2 and multi-omic extensions such as SHARE-seq and scNMT-seq, addressed the cellular heterogeneity limitation by cataloguing cell-type-specific methylation programs at unprecedented granularity, illuminating lineage trajectories, tumor cell hierarchies, and the joint regulation of chromatin accessibility, methylation, and transcription within individual nuclei [12]. Yet even single-cell resolution remains structurally blind to tissue architecture: the spatial arrangement of cells within glands, cortical layers, immune niches, and tumor microenvironmental zones is not incidental but is a primary regulatory variable. A cell’s methylation state is shaped not only by its intrinsic lineage program but by its position-specific exposure to morphogenetic gradients, paracrine signaling, extracellular matrix composition, and the metabolic microenvironment defined by local oxygen tension and nutrient availability [13]. This fundamental insight motivates the emerging paradigm of spatial methylomics: the co-measurement of DNA methylation and its functional correlates, transcriptome and chromatin accessibility, within their native tissue context at near-single-cell spatial resolution. A landmark 2025 study by Li et al. realized this vision by simultaneously profiling the DNA methylome and transcriptome from intact tissue sections at cellular resolution, generating bimodal spatial maps of mouse embryogenesis and postnatal brain development that revealed how methylation–gene expression relationships are organized along spatial axes inaccessible to dissociation-based approaches [14]. The spatial integration framework is underpinned by mechanistic interdependencies among the three principal regulatory layers. DNA methylation at enhancers and promoters modulates chromatin accessibility; accessible chromatin determines transcription factor occupancy; and transcription factor activity drives gene expression programs that reciprocally regulate the epigenetic writing and erasing machinery, a logic that recent multi-omic single-cell methods have already begun to decode [12]. Extending this integration to the spatial dimension, enabled by the convergence of spatially barcoded capture arrays, miniaturized bisulfite sequencing library protocols, and computational frameworks pioneered by spatial transcriptomics platforms (10× Genomics Visium, Slide-seq, MERFISH), now permits the inference of spatially defined gene regulatory networks that capture the positional logic of tissue organization [5]. Long-read sequencing technologies—Oxford Nanopore and PacBio HiFi—further enrich this framework by enabling direct, conversion-free detection of 5mC and 5hmC, providing haplotype-resolved spatial methylation maps that capture allele-specific imprinting and the co-methylation of distant CpGs reflecting higher-order chromatin architecture [5]. Methods achieving simultaneous resolution of chromatin accessibility, 3D genome organization, and CpG methylation in single molecules, such as SCA-seq, illustrate the depth of information now accessible within a single experimental workflow [15].

The research and clinical imperatives driving investment in spatial methylomics span oncology, neuroscience, and precision diagnostics. In cancer, the tumor microenvironment (TME) constitutes a spatially organized ecosystem where epigenetically distinct tumor subclones, cancer-associated fibroblasts, endothelial cells, and immune populations coexist in architecturally defined configurations that collectively determine therapeutic response. The progressive methylation-driven silencing of effector cytokine loci and demethylation of T cell exhaustion markers (*PDCD1*, *HAVCR2*, *LAG3*) is a spatially organized process whose anatomical correlates within tumors directly predict immunotherapy response, a relationship only resolvable through spatial epigenomics [13]. In neuroscience, the laminar and circuit-level architecture of the brain demands spatially resolved methylomics to assign cell-type- and region-specific epigenetic programs to their precise anatomical coordinates; this approach has recently been validated clinically in the NePSTA framework, a spatial transcriptomics plus graph neural network pipeline that predicts methylation-based CNS tumor subclasses from single 5 µm FFPE (formalin-fixed paraffin-embedded) tissue sections with accuracy validated across 130 participants and four medical centers [16]. More broadly, the trajectory from bulk methylation biomarkers to spatially resolved epigenomic diagnostics exemplified by the clinical translation of cfDNA methylation assays for multi-cancer early detection underscores the transformative potential of this field for disease stratification, early detection, and therapeutic monitoring at a resolution and biological specificity that transcends conventional approaches [17]. This review highlights current updates in epigenetics.

## 2. Network and Factor-Model Integration Across Omics (From Multi-Layer Omics to Methylation-Centered Disease Networks)

### 2.1. Benchmarking Integrative Analysis: Lessons from Multi-Omics Cancer Atlases and Human Epigenome Projects

Current research on integrative multi-omics analysis in oncology places increasing emphasis on epigenetics as the key to reconstructing regulatory networks that drive tumorigenesis. Single-omics approaches capture only isolated facets of gene regulation, whereas multi-omics integration, particularly through epigenomic layers such as DNA methylation and chromatin architecture, is essential for a systems-level understanding of cancer mechanisms [18,19]. Specialized databases and resources remain indispensable for these analyses. As surveyed by Das et al. [20], pan-cancer and general-purpose multi-omics platforms supply the foundational infrastructure, with TCGA delivering high-resolution epigenomic profiles including DNA methylation data generated by Illumina Infinium arrays (HM27, HM450, and EPIC) and processed as beta-values at individual CpG sites across more than 20,000 samples from 33 cancer types. This resource is complemented by the Gene Expression Omnibus (GEO), which archives a wide spectrum of epigenomic datasets derived from arrays or whole-genome bisulfite sequencing, thereby supporting cross-disease interrogation of epigenetic landscapes. These primary repositories are further augmented by dedicated annotation platforms that integrate and interpret epigenomic signals. Ensembl [21] (ENCODE, Roadmap Epigenomics, Blueprint), enabling visualization of chromatin states, histone marks, and transcription factor binding sites across more than 37,000 genomes. In parallel, FANTOM [22] focuses on non-coding regulatory elements, delivering promoter and enhancer maps through Cap Analysis of Gene Expression (CAGE), chromatin interaction data from Hi-C, and related chromatin analyses accessible via the ZENBU-Reports and fanta.bio interfaces. Single-cell resolution of epigenetic heterogeneity is advanced by the scMMO-atlas [23], which harmonizes multimodal data from over three million cells across 27 tissues with prominent inclusion of scATAC-seq for chromatin accessibility. Disease-specific portals extend this epigenomics-centric infrastructure to targeted cancer contexts. FORALL [24] offers an interactive platform for navigating multi-omics data of pediatric acute lymphoblastic leukemia cell lines, integrating epigenetic profiles with transcriptomic, proteomic, and drug sensitivity datasets to facilitate exploration of regulatory mechanisms and therapeutic responses. Similarly, MOBCdb [25] incorporates DNA methylation profiles together with clinical parameters for breast cancer subtypes, offering integrated survival analysis. DriverDBv3 [26] enables synergistic gene-pair analysis centered on DNA methylation data to refine cancer driver identification. MLOmics [27] supplies a uniformly processed, machine learning-ready compendium of 8314 samples from 32 cancer types, featuring promoter and region-level DNA methylation beta-values along with pre-built baseline models optimized for downstream integrative modeling. A structured overview of all databases and resources described above, including their omics data types, epigenomic coverage and analytical scope, is provided in Table 1.

The availability of large integrated datasets has led to the active development of multi-omics data integration methods and the need for their systematic comparison. In this regard, special attention is given to benchmarking studies aimed at the objective evaluation of the performance of various algorithms [28,29]. A significant portion of existing methods is based on joint dimensionality reduction and latent factor modeling approaches. Cantini et al. [30] benchmarked MOFA (Multi-Omics Factor Analysis), iCluster, JIVE, MCIA, RGCCA, intNMF, tICA, and scikit-fusion on TCGA data, forming a shared latent factor space reflecting joint multi-omics variation. Although all methods assume Gaussian or near-Gaussian data distributions, DNA methylation beta-values included in the TCGA omics layers are bounded to [0, 1] and frequently exhibit bimodal distributions at CpG sites, a distributional specificity that is not addressed at the preprocessing stage of this benchmark. A similar comparative analysis is presented in the work by Cai et al. [31], which investigated the MOFA2, DIABLO, iClusterPlus, iClusterBayes, and moCluster methods using Cancer Cell Line Encyclopedia data. To ensure comparability, a single downstream Random Forest model with repeated 5-fold cross-validation evaluated methods on cancer type classification accuracy and drug response prediction. The results demonstrated the advantage of the supervised DIABLO approach, whereas most unsupervised methods did not show significant superiority over baseline integration strategies, such as PCA or early feature concatenation. The performance advantage of DIABLO is partly explained by its sparse PLS-DA architecture, which optimizes inter-omics covariance in a label-guided manner without imposing distributional assumptions on individual layers, making it inherently more tolerant of the heteroscedastic and bounded nature of DNA methylation beta-values compared to the Gaussian latent variable frameworks underlying iClusterPlus and iClusterBayes. One of the key challenges in multi-omics analysis remains the high dimensionality and noise of the data, which stimulates the development of preliminary filtering and feature selection methods. Pang et al. [32] proposed Feature Set Denoising in the AttentionMOI framework, applying ANOVA, LASSO, and PCA for preprocessing feature selection. Subsequently, a neural network with an attention mechanism is used, allowing for the automatic evaluation of the contribution of different omics layers to tumor classification and survival prediction tasks. For DNA methylation data specifically, ANOVA-based filtering targeted differentially methylated CpG sites with maximal inter-group variance, while LASSO penalization addressed the extreme dimensionality of Illumina arrays, together reducing the active feature set to epigenetically informative loci prior to attention-based integration. Concurrently, methods for the deep integration of multi-omics data are being actively developed. The benchmarking analysis by Leng et al. [33] examines 16 different deep learning architectures, including variational autoencoders (VAEs). These models make it possible to identify complex nonlinear dependencies between different types of omics data by forming a shared latent representation space. However, the high predictive accuracy of such models is often accompanied by reduced interpretability and increased demands on the volume of training data. DNA methylation data were incorporated as beta-value inputs subjected to standard normalization across all 16 evaluated architectures; however, the Gaussian reconstruction likelihoods of VAE-based models represent a formal distributional mismatch with the bimodal character of CpG-level beta-values, an unresolved limitation for architecture refinement.

In response to this problem, interpretable and hybrid approaches are being developed that integrate biological knowledge into the model structure. For example, MULGONET [34] uses the Gene Ontology hierarchy to construct a neural network architecture that simultaneously predicts patient survival and identifies functional biological modules associated with tumor progression. DNA methylation signals are incorporated at the level of GO-term-associated gene sets, whereby CpG-level beta-values are aggregated into functional modules within the ontology hierarchy, contextualizing epigenetic variation within regulatory pathways and substantially reducing sensitivity to the distributional heterogeneity of individual methylation probes. A similar two-stage approach by Huang et al. [35] uses Sparse Canonical Correlation Analysis (SCCA) to identify correlated features between omics layers, followed by a neural network to model their nonlinear interactions. Critically, the SCCA stage explicitly models the chain association between DNA methylation and mRNA expression, whereby CpG methylation regulates transcription factor binding and downstream gene silencing or activation by applying FGL-SCCA with fused and graph-guided pairwise group lasso penalties to extract sparse canonical correlations between beta-values and expression profiles as the primary epigenetic feature selection step. In addition to classification and prediction tasks, integration methods are actively applied in multi-omics data clustering. The MONET algorithm [36] implements a graph-based approach to integrative clustering, forming sample modules based on heavy subgraphs in similarity networks for each omics layer. This approach accounts for the fact that cluster structure may be determined by only a portion of the available data and demonstrates robustness to missing values in TCGA datasets. Its non-parametric similarity network construction imposes no distributional assumptions on beta-values, and in the glioblastoma dataset, MONET autonomously selected DNA methylation as the sole informative omics layer across all clustering modules, reflecting the significant role of methylation status in GBM molecular stratification.

**Figure 1:**
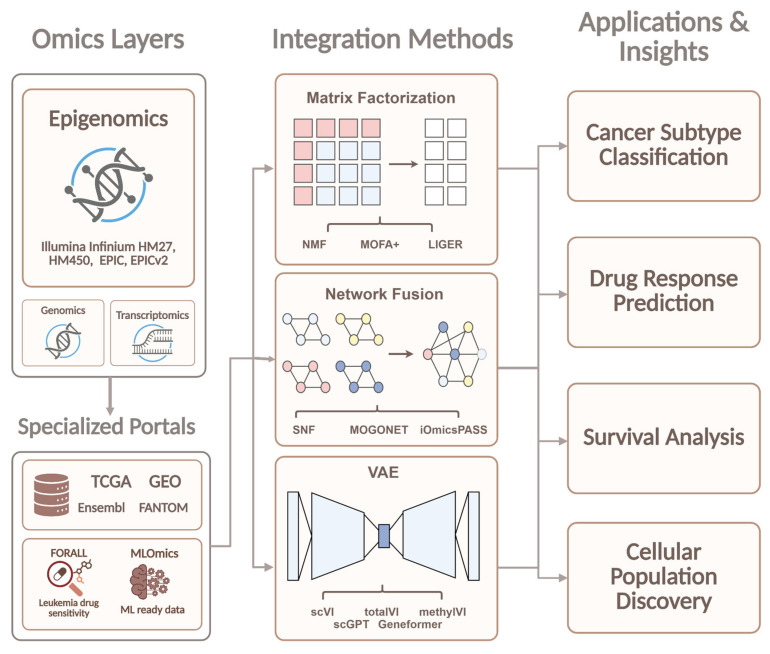
Visualization of benchmarking integration approaches for multi-omics data analysis in cancer research.

With the development of single-cell multi-omics technologies, integration methods are also being adapted for the analysis of cellular data. The SC-JNMF method [37] uses joint non-negative matrix factorization to factorize multiple feature matrices into a shared latent cell space, integrating scRNA-seq data and improving cell clustering. The non-negativity constraint of the joint NMF formulation is inherently compatible with DNA methylation beta-values, which are naturally bounded to [0， 1], suggesting methodological transferability to single-cell multi-omics protocols co-profiling chromatin accessibility or CpG methylation alongside transcriptomic data. The growing number of integration algorithms has necessitated the creation of specialized benchmarking platforms. The SurvBoard platform [38] provides a standardized system for evaluating multi-omics survival analysis models using C-index, Kaplan–Meier, and D-Calibration metrics. By explicitly standardizing preprocessing decisions, including missing CpG probe imputation strategies and normalization choices for epigenomic modalities, SurvBoard addresses a critical source of non-comparability in survival benchmarks that incorporate DNA methylation data. Furthermore, similar benchmarking studies compare single-cell integration methods (Seurat, Harmony, LIGER, Scanorama, MOFA+) based on preserving biological cell structure and correcting batch effects. Finally, a distinct area of research is the development of methods capable of working effectively with limited training data volumes. In this context, MMOSurv [39] uses meta-learning with a deep Cox survival model to transfer meta-knowledge across cancer types and adapt to small-sample tasks. DNA methylation constitutes a particularly valuable omics layer in this framework, as partially conserved pan-cancer CpG methylation patterns provide transferable epigenetic meta-knowledge that enables effective parameter initialization of the survival model even when cancer type-specific methylation training data are scarce. A conceptual scheme of the benchmarking of multi-omics data analysis methods, reflecting the main stages of comparative analysis, is presented in Figure 1.

### 2.2. Machine Learning and Deep Generative Integration Models

#### 2.2.1. Matrix Factorization Network Fusion

Modern multi-omics research frequently grapples with the “curse of dimensionality,” where thousands of biological variables generate excessive informational noise. To address this, the initial phase of analysis typically employs matrix factorization techniques, such as NMF [40] and MOFA+ [41]. These algorithms deconstruct complex methylation and expression profiles into compact “latent factors” that capture genuine biological processes rather than technical variation. As these approaches evolved, it became evident that purely mathematical compression is insufficient for deciphering functional relationships. This realization gave rise to the field of Network Fusion. Methods such as Similarity Network Fusion (SNF) [42] and MOGONET [43] transform data tables into interaction graphs, where nodes represent molecules and edges signify their regulatory connections. This shift from matrices to networks enables researchers not only to cluster cells but also to identify the core regulatory subnets governing cellular fate. Taken together, these approaches are moving from simple data compression toward biologically structured integration: factor models summarise shared variation, whereas network fusion reconstructs regulatory relationships. Their main limitation remains sensitivity to sparsity and noise, which has driven the field toward probabilistic generative models.

#### 2.2.2. Deep Learning: From Autoencoders to Foundation Models

Despite the efficacy of graph-based methods, the exponential growth of data volume necessitated the adoption of even more powerful computational architectures. Classical factorization has increasingly been supplanted by deep generative models, particularly Variational Autoencoders (VAEs). The scVI family [44] and specialized tools like methylVI [45] have revolutionized integration by learning to approximate the complex probability distributions of omics signals. This allows for the seamless merging of diverse modalities while preserving subtle biological nuances, even in the presence of significant technical noise or data sparsity.

The logical progression of this technological branch is the implementation of Transformer architectures, which now define the state-of-the-art in systems like scGPT [46] and Geneformer [47]. Trained on millions of single-cell profiles, these “Foundation Models” treat genetic sequences and methylation levels as a form of “biological language.” This paves the way for universal predictive systems capable of forecasting methylome states in a zero-shot manner. The trade-off is that predictive power increases faster than interpretability, making model explanation and biological validation a central challenge.

#### 2.2.3. Spatial and Multi-Modal Co-Profiling (Spatial and Dual-Modality Epigenomics)

The next step beyond computational integration is direct co-profiling, where two or more molecular layers are measured in the same cell or tissue section. This reduces the alignment uncertainty inherent in post hoc integration and preserves anatomical context. Spatial-DMT is a notable example, enabling joint mapping of transcriptome and DNA methylome states in intact tissue sections and thereby linking epigenetic regulation to local tissue architecture [14].

At single-cell resolution, complementary platforms capture additional regulatory dimensions. snm3C-seq connects DNA methylation with three-dimensional chromatin organisation, whereas Methyl-ATAC-seq links methylation to chromatin accessibility within the same nucleus [48,49]. These technologies make it possible to ask not only where methylation changes occur, but also how they relate to chromatin state and spatial organisation.

Interpreting such datasets requires downstream frameworks that translate multimodal signals into regulatory programs. Methods such as SCENIC+ infer regulons and cell-state transitions from integrated chromatin and expression data, providing a functional layer for co-profiled measurements [50]. Overall, the field is moving toward direct, spatially resolved measurement of regulatory layers, where methylation can be analysed not in isolation but as part of the local gene-regulatory environment.

### 2.3. Conceptual Decision Tree: How to Select an Integration Framework Based on Sample Type and Resolution

#### 2.3.1. Data Harmonization Across 450K, EPIC v1/v2, and WGBS Platforms

Aggregating training data across the 450K BeadChip, EPIC v1.0, and EPIC v2.0 arrays is a prerequisite for multi-cohort biomarker development but introduces platform-specific technical variation that can confound biological inference. A comprehensive evaluation of EPIC v2.0 by Peters et al. confirmed high reproducibility with v1.0 for shared CpG positions but identified cross-hybridization at redesigned probes introduced to replace technically suboptimal v1.0 loci as a source of systematic beta-value differences requiring explicit cross-version calibration in pooled analyses [9]. Standard preprocessing pipelines address within-platform technical variation through probe-type normalization (BMIQ for Infinium I/II imbalance; SWAN), background correction via the Noob method, which leverages out-of-band fluorescence to estimate per-sample technical background, and quantile or functional normalization [8]. Cross-platform integration with WGBS further demands statistical reconciliation of count-based fractional methylation estimates with array beta values across regions of heterogeneous sequencing depth, and careful treatment of the distinct noise distributions of the two measurement modalities [5]. A pervasive threat to cross-ethnic clinical biomarker development is the presence of single-nucleotide polymorphisms (SNPs) within or flanking Infinium probe hybridization sequences, which generate genotype-dependent fluorescence differences that masquerade as methylation variation across ancestrally diverse populations. Filtering probes at polymorphic positions using curated annotation databases such as that provided by Zhou et al. removes 37.5% of apparent meQTL-associated (Methylation quantitative trait loci) CpGs that are in fact artifactual associations driven by probe SNPs strongly differentiated between African and European ancestral populations [51], a finding underscoring the mandatory nature of SNP-probe filtering in any clinical-grade biomarker development program. meQTLs genetic variants that influence DNA methylation at specific CpGs constitute an additional source of population-specific methylation variation: a large-scale cis-meQTL mapping study in 961 African Americans from the GENOA cohort identified over 4.5 million cis-meQTLs with 45% of meCpGs harboring multiple independent meQTLs, demonstrating the complexity of genetically driven methylation variation in non-European populations and its implications for cross-ethnic model transferability [52].

#### 2.3.2. Batch Correction, Cross-Lab Calibration, and Cross-Ethnic Model Transferability

Batch effects arising from differences in bisulfite conversion efficiency, DNA input quality, scanner generation, reagent lot, and laboratory protocol represent the dominant threat to the reproducibility of methylation biomarkers across clinical sites. Ross et al. systematically characterized batch-effect sources in Illumina 450K and EPIC arrays across multiple laboratory conditions, establishing that chip position, processing date, and bisulfite conversion reagent lot are the primary technical drivers and recommending their prospective inclusion as blocking factors in multi-center study design [53]. Wang and colleagues introduced ComBat-met, a beta-regression extension of the ComBat Bayesian empirical-Bayes framework specifically adapted to the bounded [0, 1] distribution of methylation beta values, demonstrating improved statistical power for biological effect detection without inflation of false-positive rates relative to standard M-value ComBat, a distinction with direct regulatory significance for clinical classifier development [54]. For bisulfite sequencing-based assays, synthetic DNA standards with certified methylation levels spanning the full dynamic range serve as inter-laboratory calibrants, analogous to NIST Standard Reference Materials in clinical chemistry, enabling quantitative inter-site comparability essential for multi-center validation studies. Cross-ethnic model transferability remains an underappreciated but critical dimension of clinical epigenomic biomarker development with direct implications for health equity. Because meQTL allele frequencies differ across ancestral populations as documented by Shang et al. for African Americans [52] and by Li et al. for admixed populations [55], methylation classifiers trained predominantly on European-ancestry cohorts may exhibit systematic miscalibration when applied to patients of non-European ancestry, creating disparity in diagnostic performance. Li et al. further demonstrated that incorporating meQTL genetic effects into EWAS models significantly improves model fit, with approximately 20% of CpGs in the genome showing methylation levels influenced by common SNP genotypes, a proportion large enough to materially affect classifier performance across diverse populations [56]. Mitigation requires prospective enrollment of ancestrally diverse training cohorts, ancestry-aware normalization, and pre-specified subgroup performance analyses in regulatory submissions with adequate statistical power for meaningful stratified evaluation.

#### 2.3.3. A Decision Framework for Cross-Platform DNA Methylation Data Integration

**Figure 2:**
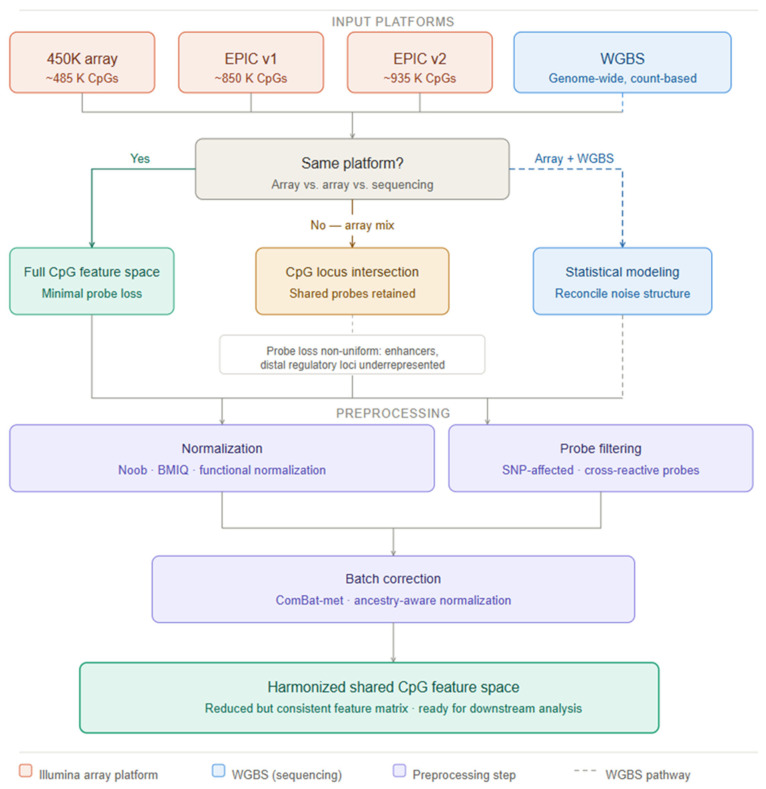
Cross-platform methylation data integration: a decision framework.

Building on the normalization and batch-correction considerations outlined above, selecting an appropriate integration strategy for multi-cohort DNA methylation data is a consequential analytical decision that determines which biological signals are preserved and whether downstream classifiers will generalize across datasets. The central constraint is platform-specific probe design: because the HumanMethylation450K and MethylationEPIC arrays interrogate overlapping but non-identical CpG loci [57,58], no universal pipeline recovers the full feature space of each contributing dataset simultaneously. The decision framework proceeds along three axes—platform homogeneity, data resolution, and the biological question under investigation. Same-platform datasets can exploit the full shared CpG space with preprocessing effort concentrated on batch correction and normalization; cross-platform integration requires restriction to the locus intersection, a reduction non-uniform across the genome since platform-specific probes are disproportionately enriched in enhancer and distal regulatory regions [8,57], systematically underrepresenting loci most relevant to gene regulatory inference. Integration of sequencing-based data, such as WGBS, introduces a further layer of complexity, as count-derived fractional methylation estimates and array beta values carry fundamentally different noise architectures requiring explicit statistical reconciliation rather than simple feature intersection [54]. Critically, the biological unit of analysis modulates tolerance for probe loss: bulk tissue integration accepts CpG intersection with relatively modest consequences, whereas cell-type-resolved or spatially localized analyses—where intra-tissue methylation heterogeneity is itself the signal—impose strict locus coverage requirements, since regulatory-region probe attrition can silently degrade deconvolution model resolution without manifesting in standard quality metrics. Functional normalization and Noob background correction [59] stabilize within-platform variation prior to cross-dataset merging, while mandatory removal of cross-reactive and SNP-affected probes [60] must precede any intersection-based alignment to prevent genotype-driven confounding from propagating into the integrated feature space. Computationally, the high dimensionality of the shared CpG matrix renders integration memory-bound, with parallel CPU execution sufficient at current dataset scales. Cross-platform methylation integration is thus most productively framed as a constrained feature-alignment problem in which heterogeneous datasets are projected onto a shared epigenomic coordinate system under simultaneous technical and biological constraints; Figure 2 formalizes this decision logic as a structured framework linking dataset characteristics to integration strategy in a reproducible and explicitly justified manner.

## 3. Long-Read, Native-DNA Methylation for Variant–Methylation Phasing

The computational frameworks surveyed in Section 2 depend on multi-omics datasets generated by sequencing platforms that, until recently, shared a fundamental constraint: short read lengths. Short-read bisulfite sequencing produces methylation averaged across cell populations in 150–300 bp fragments, preventing observation of specific CpG sites and nearby genetic variants on the same DNA molecule. Long-read sequencing (LRS) technologies, including Oxford Nanopore Technologies (ONT) and Pacific Biosciences (PacBio), address this by reading native DNA spanning tens to hundreds of kilobases, enabling simultaneous capture of sequence variants and base modifications without bisulfite conversion [61].

ONT sequencing enables the direct detection of DNA methylation from the ionic current signal of single molecules, offering a unique advantage over conventional short-read methods that require chemical conversion or immunoprecipitation [62]. This capability allows for the identification of diverse base modifications, including 5mC, 5hmC, and N6-methyldeoxyadenosine (6mA), without degrading the native DNA template [62,63]. PacBio utilizes Single Molecule Real-Time (SMRT) sequencing to detect base modifications by analyzing the kinetics of the DNA polymerase [64,65]. In this system, the timing between base incorporations is altered by the presence of methylated bases, allowing the platform to identify modifications on native DNA templates during the sequencing process [64,65]. This is often performed using Circular Consensus Sequencing to generate high-fidelity reads that provide the accuracy necessary for precise epigenetic mapping [64].

### 3.1. Direct Base Modification Calling with Long Reads

Building on the technical foundations of ONT and PacBio, the primary advantage of these LRS platforms lies in their ability to capture epigenetic information from native DNA without the biases of PCR amplification or chemical conversion [64,66]. The translation of these raw signals into accurate methylomic maps relies on advanced computational frameworks that interpret platform-specific data deviations [67,68]. Both platforms employ deep learning models to interpret platform-specific signal deviations, disruptions in ionic current for ONT and polymerase kinetics for PacBio, accordingly, achieving 5mC detection sensitivities exceeding 90% [67,69]. The performance of these signal-to-map translations has been substantiated by rigorous benchmarking, which demonstrates that the latest R10.4 nanopore flowcell chemistry and advanced base-calling algorithms achieve performance metrics comparable to SMRT sequencing and oxidative bisulfite sequencing for CpG methylation detection [65].

Based on these benchmarks, these platforms support the simultaneous detection of multiple modification types from full-length reads at single-molecule and genome-wide resolution [70]. The more recent DeepMod2 is designed to handle newer data formats such as POD5 and the latest R10 flowcell chemistries [62], and Dorado with R10.4.1 flowcells now enables direct real-time modification calling through integrated models, including Remora and Uncalled4 for refined signal alignment [71,72]. For PacBio, ccsmeth and modbamtools extract modification calls from HiFi consensus reads, with ccsmeth utilising BiGRU and attention mechanisms to achieve high correlation with bisulfite sequencing for 5mCpG detection, while ccsmethphase extends this to haplotype-resolved allele-specific methylation analysis [64,65].

Beyond modification detection per se, long-read native DNA sequencing has recently been extended to the simultaneous profiling of DNA methylation and chromatin accessibility at single-molecule resolution [5,73]. This concept was exemplified by the Direct Transposition of Native DNA approach, in which Tn5 transposase is adapted to insert sequencing adapters directly into accessible chromatin regions of native, unconverted DNA, enabling the co-readout of CpG methylation status and nucleosome occupancy from the same molecule within a unified workflow [73]. In contrast to short-read ATAC-seq or MeDIP-seq, which require separate population-level assays and inherently average signals across heterogeneous cell mixtures, DTND preserves native covalent modifications and chromatin structure over kilobase-scale fragments, allowing direct inference of the co-regulation of chromatin openness and DNA methylation on individual alleles [73,74]. This capability is particularly valuable for resolving allele-specific regulatory states in mosaic tissues, tumor subclones, and pre-malignant lesions, where subtle shifts in both methylation and accessibility may underlie emergent disease phenotypes [73].

### 3.2. Advantages of Haplotype-Aware Methylome Mapping

Beyond base modification accuracy, the primary structural advantage of LRS lies in spanning tens to hundreds of kilobases, facilitating the reconstruction of complete haplotypes and the direct observation of allele-specific methylation patterns across complex genomic loci [73,75]. Haplotype-aware methylome mapping significantly extends phase length N50 by 78–151% at phasing accuracies from 83.4% to 98.7%, surpassing the limitations of SNV-only (single nucleotide variant) strategies [76]. Longer phase blocks reduce fragmentation, preserving allele-specific regulatory signals in downstream analyses. Tools such as MethPhaser leverage heterozygous methylation patterns as additional markers across autosomes to connect phase blocks and boost overall phasing performance, particularly in medically relevant regions like the HLA locus [76]. By treating allele-specific methylation as haplotype markers, this approach resolves phase blocks across repetitive regions and structural variants inaccessible to short-read or SNV-based methods [76], reducing total phase blocks from 11,916 to 9837 and streamlining downstream analysis for more accurate identification of disease-relevant variants [76].

Such haplotype-resolved analysis is particularly critical for interrogating epigenetic heterogeneity in mosaic tissues and cancer, where allele-specific methylation events can drive differential gene expression and influence tumor progression [63,77]. Long-read technologies enable the direct phasing of single-nucleotide variants with methylation states across individual DNA molecules, allowing researchers to determine whether specific CpG sites are methylated on the same haplotype as a pathogenic mutation [65,78]. This direct correlation between genetic variants and regulatory methylation patterns on the same physical molecule is fundamental to understanding genotype–epigenotype interactions in complex diseases [79].

Targeted adaptive LRS generates chromosome-phased datasets for identifying coding and non-coding disease-causing alleles, with higher precision than short-read technologies [80]. Haplotype-aware mapping has enabled the discovery of allelically DMRs (aDMRs) in cancer-driver genes [78]. These aDMRs influence tumour progression and treatment response, and are undetectable in bulk short-read data where methylation is averaged across both alleles.

### 3.3. Duplex Sequencing, Consensus Accuracy, and Methylation Phasing

Accurate modification calling and haplotype-aware mapping, as described above, depend ultimately on the fidelity of the underlying sequencing chemistry. Two complementary strategies address this at the platform level. The latest generation of ONT flow cells, specifically the R10.4.1HD series, offers duplex sequencing capabilities where both strands of a single double-stranded DNA molecule proceed successively through a pore to generate a high-fidelity consensus sequence. This dual-strand approach achieves Q30 accuracy with a median error rate below 10^−7^, which is critical for distinguishing true biological variation from the stochastic noise typically associated with single-molecule measurements [81,82]. By capturing information from both the sense and antisense strands, duplex sequencing permits the simultaneous determination of strand modification symmetry and enables the precise phasing of single-nucleotide variants with methylation states across individual molecules [81,83]. This technical advancement effectively addresses the variable basecalling accuracy challenges noted in earlier iterations of LRS by providing a robust consensus derived from the same physical template [82,84].

**Figure 3:**
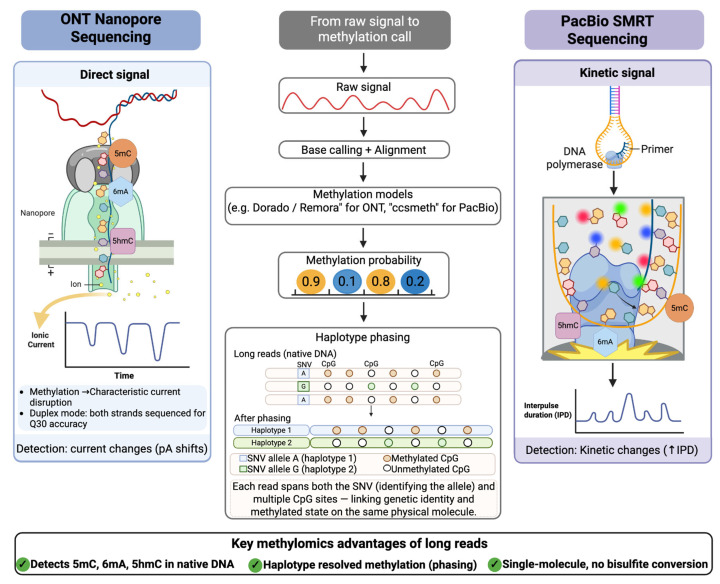
Comparison of LRS platforms: direct base modification detection and haplotype-resolved methylation phasing by LRS.

PacBio circular consensus sequencing leverages multiple polymerase passes around a circularized template to produce high-fidelity reads that reach precision and recall rates exceeding 99.91% for small variants and indels [85]. Unlike short-read platforms that require bisulfite treatment, which causes significant DNA degradation and library bias, single-molecule platforms like PacBio and ONT analyze native DNA in its original state (Figure 3) [66,86]. This preservation of the native molecule allows these technologies to resolve “NGS (next-generation sequencing) dead zones” found in highly repetitive or GC-rich genomic architectures, which were previously inaccessible to short-read whole-genome sequencing [87,88]. By avoiding chemical conversion, these platforms maintain a high correlation with traditional methylation benchmarks while delivering superior performance in identifying complex variations [64].

Beyond structural detection, the ability to phase genetic variants with methylation states enables the direct determination of parent-of-origin for de novo mutations and the diagnosis of imprinting disorders [87,88]. This precise correlation between methylation and variants facilitates the identification of epigenetic defects where the parental origin of the variant dictates its phenotypic expression [87,89]. The capacity to phase methylation across whole chromosomes allows for the identification of multi-locus imprinting disturbances, which can have diverse phenotypic consequences that are often obscured in bulk short-read data [90,91]. As these high-fidelity technologies move toward routine clinical use, they provide the diagnostic resolution needed to resolve the complex variant–methylation interactions illustrated in the case studies of Section 3.4 [92,93]. Table 2 provides a concise head-to-head comparison of ONT Duplex and PacBio HiFi CCS platforms for clinical methylation detection.

### 3.4. Case Studies in Variant–Methylation Phasing

The following examples illustrate how the variant–methylation phasing capabilities described above translate into concrete diagnostic gains across several disorder classes [89]. Congenital imprinting disorders, such as Angelman syndrome, Prader–Willi syndrome, and Russell-Silver syndrome, serve as the “gold standard” for this application [87,95]. By simultaneously performing SNV-based haplotyping and enumeration of 5mC/C-containing reads, LRS can directly visualize parent-of-origin-specific methylation patterns [88,89]. At the 15q11-q13 locus, this single assay allows for the discrimination of complex molecular causes, including large deletions, uniparental isodisomy, and heterodisomy, achieving 100% concordance with traditional molecular tests [87,96].

The same single-molecule resolution that clarifies imprinting disorders is equally applicable to pathogenic short tandem repeat expansions, which present a similar challenge: causal variants that are structurally complex and epigenetically regulated [89]. For instance, it can accurately characterize the D4Z4 macrosatellite contraction in Facioscapulohumeral Muscular Dystrophy 1 and the GGC repeat expansion in the *XYLT1* gene associated with Desbuquois dysplasia type 2 [87,89]. This capability is particularly vital in “NGS dead zones”, the regions of the genome that are repetitive, highly homologous, or GC-rich and thus remain refractory to conventional short-read analysis [87,88]. The clinical stakes of this capability are illustrated by the identification of immunodeficiency-causing variants in the *IKBKG* gene, where LRS resolved a diagnosis in a patient who had previously received a negative short-read genome sequencing result [88].

Targeted long-read approaches using adaptive sampling have further optimized these workflows, providing efficient one-step assays that identify altered methylation while informing clinical management and genetic counseling [87]. Collectively, these case studies demonstrate that native-DNA methylation phasing provides a unified diagnostic framework capable of resolving complex genetic and epigenetic interactions that were previously unresolved by short-read modalities [78,93]. The full potential of this diagnostic framework is realised when methylation phasing is integrated with three-dimensional chromatin organisation and spatial transcriptomic data, as explored in Section 3.5 [78].

### 3.5. Integration with 3D Genomics and Spatial Transcriptomics

The variant–methylation phasing and clinical diagnostic applications described above operate on linear sequence data. Layering this information onto three-dimensional chromatin architecture and spatially resolved transcriptomic maps reveals how allele-specific epigenetic states are organised in nuclear space and expressed across tissue contexts [97,98]. The convergence of long-read methylome data with high-throughput chromatin conformation maps, such as Hi-C, enables precise spatial resolution of regulatory interactions [97,99]. This approach reveals how phased genetic variants and allele-specific methylation patterns influence three-dimensional genome folding, long-range enhancer-promoter contacts, and disruptions to chromatin loops or nuclear compartments [97,99].

Integrated protocols like Methyl-HiC and sn-m3C-seq jointly profile chromatin architecture and DNA methylation at single-cell resolution [100,101], illuminating coordinated methylation between distal genomic segments proximal in 3D nuclear space [101,102]. This integration is valuable for imprinting disorders with allele-specific methylation driving parent-of-origin expression [90,91] and extends variant–methylation phasing with 3D maps and spatial transcriptomics, revealing allele-specific regulation in mosaic tissues and tumors. Ultimately, combining long-read methylome data with chromatin maps and spatial transcriptomics yields a spatially resolved view of gene regulation essential for complex diseases.

In summary, Section 3 has demonstrated how long-read sequencing enables precise variant–methylation phasing for detecting complex structural variations, diagnosing imprinting disorders and repeat expansions, and integrating with 3D chromatin architecture and spatial transcriptomics to reveal allele-specific regulation in nuclear space and mosaic tissues. These tissue- and single-cell-based advances, particularly those enabled by long-read sequencing for resolving allele-specific regulation in mosaic tissues, are complemented by non-invasive cell-free DNA (cfDNA) analysis, with the following chapter expanding on how bloodstream-shed fragments retain tissue-of-origin-specific methylation signatures to deconvolve cellular contributions and disease states without biopsy.

## 4. Cell-Free DNA Methylation Deconvolution

Cells undergoing apoptosis, necrosis, or active secretion release DNA fragments into the bloodstream. These cfDNA molecules retain tissue-specific methylation patterns from their originating cells, providing a molecular fingerprint [103]. This stable tissue-specificity across individuals and disease states makes cfDNA methylation a powerful non-invasive readout for identifying damaged, inflamed, or malignantly transformed tissues, their proportions, and treatment responses [104,105]. Multimodal epigenetic sequencing analysis simultaneously captures cfDNA methylation with nucleosome occupancy and fragmentation patterns, enhancing cancer detection beyond traditional markers [106].

### 4.1. Advances in cfDNA Methylation Profiling

Tumor-derived cfDNA carries cancer-specific methylation signatures that enable non-invasive biomarker discovery and therapeutic monitoring [107]. Techniques like cfMeDIP-seq use antibody enrichment for genome-wide profiling with low DNA input [108], while specialized amplification methods can detect hypermethylated CpG islands in samples with very low DNA abundance [109]. Traditional whole-genome bisulfite sequencing often degrades DNA, causing biased coverage and higher costs [106]. Consequently, bisulfite-free enzymatic methods like EM-seq and TAPS have emerged to reduce DNA damage and preserve fragmentation patterns [106]. By utilizing *TET2* and *APOBEC3A*, these approaches profile methylation from picogram quantities, allowing the integration of methylation data with fragment size and nucleosome positioning to enhance minimal residual disease detection [110,111,112].

Complementary targeted methods offer various trade-offs: restriction enzymes and locus-specific PCR provide high sensitivity for specific loci [113], while microarrays offer single-base resolution but cannot link methylation states across a single molecule [105]. Capture strategies, such as molecular-inversion probes, can target thousands of regions from low input, though the high-input requirements of some platforms limit their utility with clinical samples [94,114]. Native nanopore sequencing offers a bisulfite-free alternative, detecting 5mC and fragmentation signatures directly via ionic current changes [110,115]. This preserves DNA integrity and cell-of-origin information even at shallow coverage, making it attractive for rapid clinical monitoring [116,117,118].

However, applying long-read platforms to cfDNA is complicated by the short length of circulating fragments [119]. While Rolling Circle Amplification can improve mutation detection sensitivity, it is unsuitable for methylation analysis as it erases epigenetic features [119]. Despite these hurdles, optimized nanopore pipelines show promise for low-input samples, such as brain tumors, where rapid turnaround and decentralized testing are critical [120,121]. The diagnostic potential of these datasets relies on computational tools to decompose complex cfDNA signals into cell-type contributions, which are addressed in the following section.

### 4.2. Deconvolution Algorithms for Tissue-of-Origin and Immune Cell Tracing

**Figure 4:**
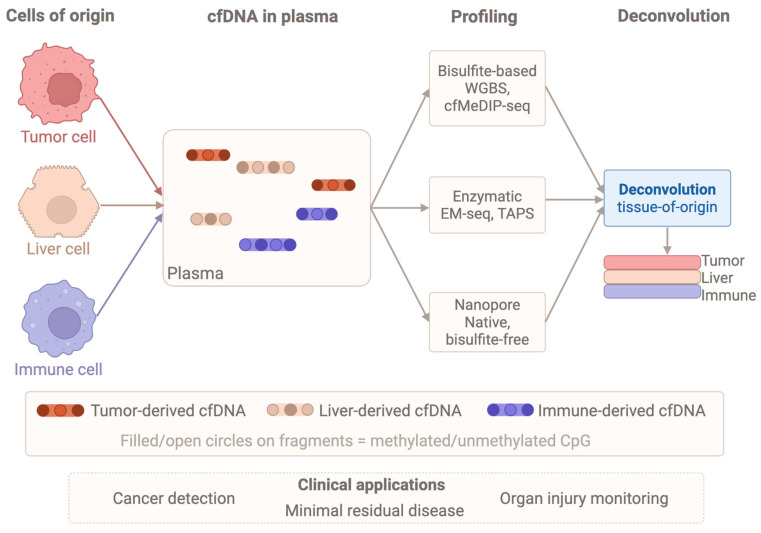
Cell-free DNA methylation profiling: tissue of origin to clinical readout.

Deconvolution approaches leverage reference methylome atlases, such as those from Illumina HM450k arrays, to estimate cellular proportions and identify tissue-of-origin for ctDNA (Figure 4) fragments, though these arrays cover only 10–15% of cell type-specific methylation markers [117]. Comprehensive WGBS-based atlases have expanded profiling to the entire genome and increased reference tissue types to 39 [119]. These atlases enable inference of tissue origins of cfDNA in a specific and sensitive manner [122].

Algorithms range from reference-based methods requiring methylation atlases from known cell types to estimate fractions via non-negative least squares, to reference-free methods inferring cell types from bulk data [123], with performance heavily influenced by sequencing depth and atlas completeness [104,117]. Computational strategies have evolved to exploit single-molecule methylation haplotypes, with models like CelFiE-ISH demonstrating 30% better accuracy than traditional tools by analyzing multi-CpG patterns on individual DNA molecules [123]. These haplotype-based methods resolve cellular mixtures with greater precision and enable more sensitive detection of rare cell types [115,123].

Marker selection is critical for maximizing deconvolution accuracy, as frameworks such as CP/QP, support vector regression, and robust partial regression demonstrate comparable precision when provided with well-curated CpG loci [124,125]. Using a defined subset of informative sites outperforms approaches that include all sites, as non-differentially methylated ones add mostly noise [122]. These strategies use CpG methylation beta values to estimate cell proportions, with CelFiE-ISH incorporating within-read haplotypes for sensitive rare cell detection and an order-of-magnitude improvement in cfDNA tissue-of-origin accuracy versus traditional CpG-site methods [119,123]. Accuracy has been validated using artificial mixtures and whole-blood DNA methylation with known cellular composition from flow cytometry [124].

Fragment length, end motif patterns, and broader fragmentomic features provide complementary non-mutational signatures for tissue-of-origin inference [126]. Long-read sequencing enables single-molecule methylation analysis of long cfDNA fragments for tissue-of-origin determination, with promise in prenatal and cancer testing [126]. Multi-modal approaches integrating methylation with copy number alterations and fragmentomic patterns outperform single-feature classifiers for multi-cancer early detection [111,127].

Performance remains highly dependent on reference library complexity and marker selection, with benchmarking studies emphasizing tailored configurations for specific assays and depths [128,129]. Non-genetic approaches, particularly methylation-based ones, overcome genetic tracing limitations in settings without well-established associations [130,131]. While these deconvolution algorithms provide high-resolution insights into cfDNA sources, their translation to clinical diagnostics requires addressing analytical validation, pre-analytical standardization, and logistical hurdles, as detailed in the next section on clinical translation.

Performance of cfDNA deconvolution algorithms varies substantially across tissue types and disease contexts, and synthesis of benchmarking data is essential for defining the clinical operating boundaries of these methods [104]. In oncology, deconvolution sensitivity is highest for hematologic malignancies and advanced solid tumors, where circulating tumor DNA (ctDNA) fractions typically reach 1–10% of total cfDNA and cell-type-specific methylation markers provide a strong discriminatory signal [132,133]. By contrast, in early-stage solid tumors, ctDNA fractions may fall below 0.1%, approaching the practical detection limit of most reference-based algorithms; multi-CpG haplotype models that exploit within-read methylation co-occurrence patterns can improve sensitivity at these low fractions by approximately an order of magnitude compared with single-CpG approaches [123,133]. For immune cell subsets such as CD4^+^ and regulatory T cells, monocytes, and neutrophils, peripheral blood deconvolution achieves near-linear agreement with flow cytometry (R^2^ often > 0.9) when comprehensive WGBS-based reference atlases are employed, underscoring the importance of reference depth and cell-type coverage [104,122,134]. Organ-specific injury applications show that plasma methylation deconvolution can detect tissue contributions as low as 0.1–1% of total cfDNA, with clinical validation in acute myocardial infarction and solid organ transplantation, where cardiomyocyte- or graft-derived methylation signatures precede conventional biochemical markers [135,136,137]. The principal detection limits of current algorithms arise from three factors: incomplete reference atlases that under-represent rare or tissue-resident cell populations [104], sequencing depth constraints, as accurate resolution of cell types contributing < 5% of total cfDNA typically requires either >30 times genome-wide coverage or equivalent targeted enrichment [104], and confounding from clonal haematopoiesis of indeterminate potential, which introduces methylation signals from expanded leukocyte clones that can masquerade as low-level tumor- or injury-derived cfDNA and therefore necessitates dedicated filtering strategies [138,139].

### 4.3. Clinical Translation of cfDNA Methylation

cfDNA methylation deconvolution has immediate clinical utility in oncology, where cfMeDIP-seq and targeted methylation assays detect tumor-derived fragments with sufficient sensitivity for minimal residual disease monitoring, treatment response assessment, and early relapse detection [115,119]. Integrative nanopore sequencing of cfDNA methylation and copy number variation enhances detection sensitivity, identifying brain cancer cases missed by CNV analysis alone [119]. In transplantation medicine, cfDNA methylation profiling non-invasively detects organ-specific injury and rejection by quantifying donor-derived DNA in recipients’ plasma [128]. Similarly, in autoimmune and neurodegenerative diseases, plasma epigenetic fingerprints reveal disease-specific tissue damage and immune activation that conventional biomarkers struggle to assess [128,140].

Despite accelerating translation driven by evolving procedures, bioinformatics, and machine learning [141], significant analytical challenges remain. The major challenge is low and variable ctDNA abundance relative to normal cfDNA, necessitating high-depth sequencing that elevates costs and risks false negatives in single-mutation detection, making cancer-specific methylation or fragmentation patterns essential for diagnostic accuracy [142,143]. Inter-individual and inter-batch variability in cfDNA yield introduces additional inconsistency [144]. Poor specificity of screening tests would result in unnecessary invasive evaluations, while molecular intratumoral heterogeneity represents a clinical and technical challenge for epigenetic biomarker translation [145].

Pre-analytical sample handling is a particularly consequential source of variability, accounting for 46–68% of errors in liquid biopsy workflows [146,147]. The International Society of Liquid Biopsy recently issued minimal quality control requirements for ctDNA analysis, stressing harmonization across pre-analytical and analytical phases [148]. Optimized pre-analytical steps, such as double plasma centrifugation and specialized blood collection tubes, can reduce wild-type DNA background and provide reliable results [109]. Blood samples in standard EDTA tubes require processing within 6 h to prevent leukocyte lysis and ctDNA contamination [146], whereas specialized tubes containing cell-stabilizing and nuclease-inhibiting cocktails extend storage time to up to 14 days without controlled environmental conditions [149,150]. This preservation of sample integrity is essential for ensuring that observed ctDNA variations reflect biological changes rather than handling artifacts [109].

In summary, Chapter 4 has detailed the evolution of cfDNA methylomics from simple mutation detection to high-resolution, tissue-of-origin mapping. Technological innovations, such as enzymatic and nanopore sequencing, now permit single-molecule, haplotype-aware profiling that avoids the degradation typical of bisulfite methods. These high-fidelity signals empower deconvolution algorithms to resolve rare cell populations with high sensitivity for applications in oncology and organ health. However, the clinical utility of these signatures depends on standardized pre-analytical protocols to manage low ctDNA abundance and sample variability. As these methodologies move from the laboratory to the clinic, they must satisfy the stringent requirements of regulatory and analytical validation. Chapter 5 explores this transition, detailing the regulatory roadmap and examining the landmark FDA-cleared methylation assays that currently define the clinical landscape.

## 5. Clinical Translation and Regulatory Roadmap

The translation of methylomic discoveries into clinically deployed diagnostics requires navigating a structured hierarchy of analytical validation, regulatory compliance, and post-market surveillance. This section delineates each layer of that roadmap and grounds it in the two most advanced exemplars of methylation-based clinical diagnostics currently cleared by the US FDA.

### 5.1. Framework for Clinical Assay Validation and Regulatory Pathways

Clinical methylation assay development is governed by a layered architecture of international quality standards and jurisdiction-specific regulatory requirements. ISO 15189:2022 [151], the fourth edition of the international standard for medical laboratory quality and competence, updated in December 2022 to align with ISO/IEC 17025:2017 [152] and to strengthen risk management requirements through explicit referencing of ISO 22367 [153], defines the quality management system, personnel competency, method validation, and pre- and post-analytical process requirements that underpin laboratory accreditation globally [151]. ISO 20395 [154] provides specific requirements for the validation of nucleic acid quantification methods with direct applicability to bisulfite sequencing and digital PCR-based methylation assays, while ISO 14971 [155] governs product risk management across the full device lifecycle. In the European Union, the In Vitro Diagnostic Regulation (EU IVDR 2017/746 [156]) fully replaced the IVDD in May 2022 [157,158]. For Class C devices, including cancer screening assays and companion diagnostics, it mandates Notified Body review of Technical Documentation, Clinical Performance Studies conducted under Common Specifications, and post-market clinical follow-up (PMCF) [157,158]. Class D devices face the most stringent requirements, including EU Reference Laboratory Scientific Opinion [157]. In the United States, the FDA’s 2024 finalization of a rule phasing out enforcement discretion for laboratory-developed tests (LDTs) subjects all clinical methylation assays, whether offered as LDTs or commercial IVDs, to premarket review [158,159]. Novel assays without a predicate may pursue De Novo review; the Breakthrough Device Designation provides expedited development interaction for assays addressing unmet clinical need, as has been applied to several cfDNA methylation tests for early cancer detection [158]. These international differences extend to data handling; the US regulatory framework, governed by HIPAA, emphasizes data de-identification and secure storage for research flexibility, whereas the EU’s GDPR establishes more stringent requirements for the handling of genomic and biomarker data [160].

Analytical validation of a clinical methylation assay encompasses: accuracy against a reference method (WGBS or digital PCR); precision (repeatability, intermediate precision across days, operators, reagent lots); analytical sensitivity (limit of blank, limit of detection, limit of quantification at target CpG positions); specificity and interferant evaluation; linearity and dynamic range; and sample-type equivalence and specimen stability under clinically relevant pre-analytical conditions. Clinical validation requires demonstration of assay performance in the intended-use population across relevant subgroups, including age, sex, ancestry, and comorbidities, with pre-specified performance thresholds and powered analyses for each subgroup [151,161].

These validation standards are reflected in existing approvals, such as the FDA approving several single-gene and multigene assays to detect genetic alterations in plasma cfDNA for use as companion diagnostics, including the Cobas EGFR mutation assay and FoundationOne Liquid CDx [162,163]. Achieving and sustaining such approvals requires robust evidence of analytical validity, including the demonstration of sensitivity, specificity, and reproducibility under conditions that simulate clinical settings [164]. Analytical validation studies of targeted methylation-based multi-cancer early detection tests have characterized sensitivity with respect to tumor content, determining limits of detection as low as 0.07% to 0.17% across tumor types while maintaining test specificity at 99.3% [164]. Technical challenges, including low tumour DNA shedding and clonal haematopoiesis of indeterminate potential, continue to limit usage, underscoring the need for robust validation schemes and standardised operating procedures to support ongoing regulatory efforts [162,165,166,167].

### 5.2. FDA-Cleared Methylation-Based Case Studies

#### 5.2.1. Cologuard: Multi-Target Stool DNA Methylation for Colorectal Cancer Screening

Cologuard (Exact Sciences Corporation) became the first FDA-cleared multi-target stool DNA (mt-sDNA) test for colorectal cancer (CRC) screening in average-risk adults [168], subsequently relabeled to lower the eligible age to ≥45 years in alignment with updated USPSTF recommendations. The methylation component interrogates promoter hypermethylation of two loci, *NDRG4* (N-Myc downstream-regulated gene 4) and *BMP3* (bone morphogenetic protein 3), selected through systematic methylome-wide screening of paired CRC tumor and normal colonic epithelium for large effect size, low background methylation in non-neoplastic stool DNA, and reliable quantitative detection by qMSP following bisulfite conversion [169]. These methylation markers are integrated with KRAS mutation analysis and fecal hemoglobin immunoassay into an algorithmic composite score, exploiting the complementary biological mechanisms of epigenetic silencing and somatic mutation in colorectal carcinogenesis.

The pivotal DeeP-C clinical trial demonstrated 92.3% sensitivity for CRC (versus 73.8% for fecal immunochemical testing, FIT) and 42.4% sensitivity for advanced precancerous lesions (versus 23.8% for FIT), with 86.6% specificity for non-neoplastic colonoscopy findings [169]. A comprehensive assessment of DNA methylation biomarkers in CRC by Fatemi et al. contextualized Cologuard’s targets within the broader landscape of CRC methylation biology, noting that *NDRG4* and *BMP3* represent early and frequent silencing events detectable across the adenoma-to-carcinoma sequence, supporting their utility for pre-malignant lesion detection [170]. The next-generation mt-sDNA test (Cologuard v2.0) incorporates a refined molecular panel evaluated in the prospective BLUE-C trial (>20,000 participants), achieving 94% CRC sensitivity at 91% specificity, outperforming both the original Cologuard and FIT, and has been submitted for FDA review [171].

#### 5.2.2. Guardant Shield: Cell-Free DNA Methylation for Blood-Based CRC Screening

Guardant Shield (Guardant Health) received FDA approval on 29 July 2024, as the first blood-based CRC screening test approved as a primary screening option for average-risk adults aged over 45 years and the first to qualify for Medicare coverage [172]. The assay deploys targeted methylation partitioning of circulating cfDNA (mp-cfDNA), enriching neoplasm-associated epigenomic alterations through proprietary probe capture, and integrating methylation pattern signals with cfDNA fragment-size features via machine learning classification [173]. This platform architecture, combining methylation biomarker enrichment with multivariate algorithmic classification, represents a methodological evolution over simpler qMSP-based detection toward the comprehensive liquid biopsy paradigm that underlies next-generation multi-cancer early detection tests. FDA approval was based on the ECLIPSE registrational study (NCT04136002), among 7861 evaluable average-risk adults across over 200 US sites, Chung et al. reported 83.1% sensitivity for CRC (95% CI 72.2–90.3%), 13.2% sensitivity for advanced precancerous lesions, and 89.6% specificity for non-advanced neoplasia [174]. The FDA Advisory Committee voted 8-to-1 for safety and 6-to-3 for efficacy in May 2024, recognizing that benefits outweigh risks given the substantial unmet clinical need in a population where over 50 million screening-eligible Americans remain unscreened [172]. The broader translational context is defined by the GRAIL Galleri test, which applies cfDNA methylation sequencing using targeted methylation-based enrichment analogous to Guardant Shield but at a multi-cancer scale to detect over 50 cancer types from a single blood draw, with tissue-of-origin prediction based on tissue-specific methylation signatures [175]. Clinical validation via the PATHFINDER prospective cohort study (6662 participants) demonstrated overall sensitivity of 29.9% for any cancer and 38.3% for the twelve high-mortality cancers with the greatest unmet screening need [176]. The landmark NHS-Galleri trial, a randomized controlled study of 142,000 demographically representative participants aged 50–77 enrolled in England’s National Health Service across three annual screening rounds (2021–2024), published topline results in February 2026. While the primary endpoint of a statistically significant combined Stage III–IV reduction was not met, the trial demonstrated a reduction in Stage IV diagnoses, increased Stage I and II detection for several deadly cancer types, and a four-fold increase in overall cancer detection rate in the intervention arm [177], providing the strongest real-world evidence to date that MCED (multi-cancer early detection) screening can favorably shift the stage distribution of cancer diagnoses at a population level. These results, together with those from the ongoing PATHFINDER 2 study (NCT05155605; 35,000 participants), are informing Galleri’s FDA submission and will collectively shape the regulatory framework for the next generation of spatially resolved epigenomic diagnostics [175,176].

Taken together, the translational arcs of Cologuard and Guardant Shield illuminate the essential principles governing successful clinical methylation assay development: rigorous, mechanism-informed biomarker discovery anchored in high-throughput methylome profiling; large-scale prospective clinical validation in demographically diverse populations with pre-specified performance thresholds; transparent multi-cohort analytical validation meeting ISO and CLIA standards; proactive regulatory engagement throughout the development program; and iterative post-market improvement. These principles will define the translational pathway for the next generation of spatially resolved, cell-type-specific methylation biomarkers, from epigenomic discovery to clinical translation.

## 6. Methylomics in Diseases

### 6.1. Cancer Methylomics

Tumors are not uniform cell populations; instead, they tend to consist of multiple epigenetically distinct cell groups. Evidence from multiregion DNA methylation studies suggests that different epigenetic subclones can coexist within the same tumor. In many cases, a higher degree of methylation heterogeneity within a tumor has been associated with more advanced disease and less favorable clinical outcomes [178,179]. These subclones are not randomly distributed. Instead, they tend to localize within distinct anatomical and functional niches, such as invasive fronts, hypoxic regions, and perivascular zones, where local microenvironmental conditions influence proliferation, cellular plasticity, and resistance to therapy [180]. At the same time, spatial variation in methylation is closely tied to immune evasion. Changes in chromatin structure at tumor–immune interfaces, together with localized immunosuppressive reprogramming, point to the idea that not all tumor regions behave the same. Some areas appear to be more effective at avoiding immune surveillance than others [181,182]. This suggests that the spatial organization of the methylome is not merely descriptive. It likely has a functional role, influencing how tumors evolve, spread, and respond to treatment.

Comparative and multiregion methylomic studies across different cancers also highlight how variable tumor epigenomes can be. Glioblastoma provides a clear example. Whole-genome methylation profiling at single-base resolution has shown higher levels of 5mC in tumor cores (63.5%) than in adjacent margins (52.1%), along with a marked reduction in 5hmC (1.6% versus 17.5%). These differences are often interpreted as a sign of impaired TET-mediated oxidation and reflect substantial epigenetic divergence between spatial tumor compartments [183]. Further multi-sector analyses suggest that multiple methylation subtypes may coexist within a single tumor. This raises an obvious question: how representative is a single biopsy for molecular classification in such a context? [178,184]. Across different cancer types, another recurring pattern is the hypomethylation of genes involved in invasion and epithelial-to-mesenchymal transition (EMT). The fact that this pattern appears repeatedly suggests that tumor invasiveness may, at least in part, be driven by shared methylation-associated mechanisms rather than entirely tumor-specific processes [185,186].

The tumor methylome is also far from static. It is continuously reshaped through interactions with the TME. Within this setting, DNA methylation contributes to the regulation of immune cell differentiation and function, influencing how immune cells behave in and around the tumor. For example, methylation-dependent processes influence CD4+ T-cell polarization toward either effector (Th1) or regulatory (Treg) states, as well as macrophage M1/M2 balance and dendritic cell maturation, largely through lineage-specific remodeling at promoters and enhancers [187]. At the same time, *DNMT3A*-driven de novo methylation contributes to the stabilization of T-cell exhaustion programs. At the same time, aberrant methylation in tumor cells can interfere with antigen presentation by silencing MHC class I genes and tumor-associated antigens, which ultimately promotes immune evasion [188]. That said, these epigenetic states are not necessarily permanent. Both experimental and clinical observations suggest that they can be modulated pharmacologically. In some settings, this has been associated with partial reversal of T-cell exhaustion, activation of viral mimicry through derepression of transposable elements, and increased immune-cell infiltration into the TME. Together, these effects provide a mechanistic rationale for combining epigenetic therapies with immune checkpoint inhibitors [187,188].

The balance between 5-methylcytosine (5mC) and 5-hydroxymethylcytosine (5hmC), regulated by TET-family dioxygenases, represents another layer of control in immune cell differentiation and function [189]. In tumors, this balance is often disrupted, and both cancer cells and infiltrating immune cells undergo substantial methylome remodeling. For example, hypermethylation at promoters of tumor suppressor and antigen-presentation genes can limit immune recognition. At the same time, epigenetic changes in tumor-associated macrophages, cytotoxic T cells, and dendritic cells tend to reinforce immunosuppressive states [188,190]. Importantly, these processes are interconnected. DNA methylation does not act alone, but works alongside histone modifications and higher-order chromatin organization to shape immune responses. This broader view has contributed to growing interest in combined epigenetic–immunologic therapeutic strategies [191]. Supporting this idea, clinical studies have shown that *DNMT* and HDAC inhibitors can increase tumor immunogenicity, reactivate viral mimicry pathways, and partially reverse T-cell exhaustion in cancers such as melanoma, non-small cell lung cancer, and renal cell carcinoma [192].

Epigenetic reprogramming is increasingly recognized as an important contributor to therapeutic resistance. One mechanism that has been repeatedly implicated in therapeutic resistance is promoter hypermethylation. This can lead to silencing of tumor suppressor genes and genes involved in drug response, including *MLH1*, *BRCA1*, and *MGMT*. As a consequence, pathways such as mismatch repair and homologous recombination become less effective, and tumor sensitivity to alkylating agents is reduced [193]. Genome-wide methylation analyses across solid tumors, including breast, colorectal, and glioblastoma, have revealed broad remodeling of the methylome in the context of treatment exposure and acquired resistance [194]. These changes are rarely isolated. They tend to occur alongside shifts in histone modifications and three-dimensional chromatin organization, which together allow tumor cells to better tolerate therapeutic stress [195]. A key difference from genetic alterations is that epigenetic changes are potentially reversible.

This has prompted increasing interest in the use of DNA methyltransferase inhibitors, particularly as a way to delay resistance or restore treatment sensitivity [192,195]. Integrating methylomic data with genomic, transcriptomic, and proteomic layers has made it easier to identify epigenetic alterations that are likely to have functional consequences. Even so, a fundamental challenge remains: separating true driver methylation events from passenger changes that arise in response to cellular stress or microenvironmental factors [196]. Large-scale pan-cancer studies that combine multiple data types have started to address this issue. In particular, they have identified cis-acting methylation events that can directly influence transcription and, in some cases, translation. This provides a more nuanced view of how epigenetic regulation contributes to tumor identity, cell-of-origin effects, and therapeutic vulnerability [197]. Another useful approach has been the integration of methylation quantitative trait locus (mQTL) analysis. By linking germline variation to local methylation patterns and downstream gene expression, these studies have identified thousands of CpG-gene-cancer associations across different tumor types [198].

#### Methylation-Based Cancer Detection: From Tissue Biopsy to Liquid Biopsy

The transition from invasive tissue sampling to liquid biopsy relies on the early detection of promoter hypermethylation in tumor suppressor genes. Aberrant silencing of genes such as *RASSF1A*, *BRCA1*, *VHL*, and *CDKN2A* disrupts critical pathways in apoptosis and DNA repair, providing stable biomarkers even in early-stage malignancies [199]. A landmark in clinical implementation is the FDA-approved Epi proColon assay, which targets methylated *SEPT9* (mSEPT9) in plasma, demonstrating up to 75% sensitivity and 96% specificity for colorectal cancer [199,200].

Advanced liquid biopsy platforms now extend beyond single-gene targets to genome-wide signatures. Profiling of 5hmC in cfDNA has emerged as a robust method for identifying tumor-specific patterns and tracking disease progression [201,202,203]. Furthermore, multi-cancer early detection assays utilize machine learning to classify signals across over 50 cancer types. Large-scale clinical evaluations, such as the CCGA study, reported 99.3% specificity and a 93% accuracy rate in predicting the tissue of origin [204]. These findings were further validated by the prospective PATHFINDER study, which demonstrated a positive predictive value of 43% in a real-world screening population [175,176]. Building on these clinical validations, targeted panels covering > 10,000 CpG sites can detect aberrations up to 5 years before diagnosis, while large-scale classifiers across 100,000 genomic regions achieve 90% tissue-of-origin accuracy [110]. Multimodal assays that simultaneously profile methylomics, fragmentomics, copy number alterations, and end motifs in a single workflow, such as SPOT-MAS, have been developed to address the signal-to-noise limitations inherent in ctDNA-based assays [142].

Methylation signatures are particularly effective in diagnosing cancers of unknown primary. Unlike mutation-based assays, methylation classifiers like CUPiD leverage lineage-specific epigenetic memory, achieving 96.8% accuracy in tissue-of-origin prediction [175,205]. This capability, combined with longitudinal profiling, allows for real-time monitoring of therapeutic response and the detection of resistance-associated epigenetic shifts, as observed in metastatic prostate and colorectal cancers [206,207,208].

Despite these milestones, the clinical utility of cfDNA methylation remains constrained by low sensitivity in Stage I disease due to minimal DNA shedding [175]. Pre-analytical standardisation, discussed in Section 5.2, remains essential for reproducible results across institutions. Addressing the current bias toward European-ancestry populations in reference datasets also remains a critical priority to ensure that MCED advancements do not exacerbate healthcare disparities [209,210].

The NHS-Galleri trial, a large prospective study of approximately 142,000 participants aged 50–77 undergoing three rounds of multi-cancer early detection screening, reported topline results in early 2026 [211,212]. Although the primary endpoint of a statistically significant reduction in combined Stage III/IV incidence was not met in the intention-to-treat analysis, several clinically relevant secondary outcomes were observed, including a significant reduction in Stage IV diagnoses and increased Stage I–II detection across high-mortality cancers such as lung, colorectal, and head and neck malignancies [211,212]. The intervention arm showed roughly a four-fold increase in overall cancer detection compared with usual care, with a positive predictive value around 43%, meaning that about 43 of every 100 positive Galleri tests corresponded to a confirmed cancer diagnosis [211,212]. Taken together, these metrics currently represent the largest prospective evaluation of cfDNA methylation-based multi-cancer early detection and define the benchmark performance against which next-generation screening assays are being compared [211,212].

### 6.2. Cardiovascular and Metabolic Diseases

DNA methylation also plays a central role in vascular epigenetic regulation in atherosclerosis. In endothelial cells, hypermethylation of the NOS3 promoter leads to reduced eNOS expression, impairing nitric oxide production and contributing to endothelial dysfunction. At the same time, methylation-dependent control of adhesion molecules such as VCAM-1 and ICAM-1 promotes leukocyte recruitment and vascular inflammation [213,214]. In vascular smooth muscle cells, aberrant methylation is associated with a shift from a contractile to a synthetic phenotype, a key step in the development of neointimal hyperplasia. This process appears to involve coordinated activity of *DNMT1* and *TET2*, linking methylation dynamics to cellular plasticity [214]. More broadly, genome-wide methylation profiling of atherosclerotic tissues has identified disease-specific epigenetic signatures that involve both endothelial and smooth muscle pathways. This points to the possibility that DNA methylation is not only a marker of disease but may also represent a potential therapeutic target in vascular pathology [215,216].

A related concept emerges in diabetes, where persistent changes in DNA methylation are believed to contribute to what is often referred to as metabolic memory. In this context, vascular complications may continue to develop even after glycemic control has been achieved [217]. Findings from the DCCT/EDIC cohort provide strong support for this concept, showing sustained hypomethylation of *TXNIP* over extended periods and linking these long-term epigenetic changes to vascular outcomes [218]. Similar observations have been reported in type 2 diabetes, where methylation patterns of *TXNIP* and ABCG1 have emerged as consistent indicators of metabolic dysfunction and increased cardiovascular risk [219]. In addition, hyperglycemia-induced methylation changes in *CXCR4* within progenitor cells have been associated with impaired vascular repair, offering more direct mechanistic insight into how epigenetic memory may contribute to disease progression [220]. cfDNA methylation analysis is also being explored as a non-invasive approach for cardiovascular disease assessment. One example is the detection of cardiomyocyte-specific methylation signatures, such as unmethylated CpG sites at the *FAM101A* locus, which can indicate myocardial injury and correlate with established clinical biomarkers [137,221]. Beyond individual markers, genome-wide cfDNA methylation profiling provides a broader view by enabling stratification of acute coronary syndromes and estimation of tissue-specific contributions, including signals from cardiomyocytes and immune cells [222]. Overall, this suggests that cfDNA methylation profiling may offer a practical way to monitor cardiovascular tissue damage in real time [223].

Epigenetic clocks based on DNA methylation offer a way to quantify biological aging and its relationship to cardiovascular risk. Among these, GrimAge has shown strong predictive performance for outcomes such as coronary heart disease and cardiovascular mortality [224,225]. Data from large cohort studies indicate that epigenetic age acceleration may partially mediate the effects of traditional risk factors on cardiac structure and function [226]. At the same time, longitudinal epigenome-wide association studies (EWAS) have identified CpG sites whose methylation levels are associated with future risk of myocardial infarction and coronary disease, with some evidence suggesting a causal component [227]. Together, these findings underscore that DNA methylation represents both a mechanistic intermediary and a clinically informative biomarker linking cumulative cardiovascular risk exposure to vascular aging and adverse cardiac outcomes.

### 6.3. Neurological and Other Disorders

DNA methylation patterns in the brain show marked regional variability across neurological disorders. In Alzheimer’s disease, differential methylation has been reported at several loci, including *ANK1*, *BIN1*, and *RHBDF2*. Among these, hypermethylation of *ANK1* has been most consistently observed across independent cohorts and appears early in disease progression [228]. In Parkinson’s disease, hypomethylation within intron 1 of *SNCA* has been associated with increased gene expression, providing a direct link between epigenetic dysregulation and α-synuclein pathology [229]. In autism spectrum disorder, both shared and region-specific methylation changes have been identified, affecting pathways related to immune function and synaptic regulation. These patterns are generally interpreted as reflecting a broader disruption of neuronal regulatory networks [230,231]. Recent advances in single-cell and spatial methylomic technologies have significantly improved our understanding of brain epigenomic organization. High-resolution datasets now point to a diverse range of neuronal subtypes, each characterized by distinct methylation signatures. They also highlight a close interplay between DNA methylation, chromatin architecture, and gene regulation [3,232,233]. These approaches make it possible to examine disease-associated epigenetic changes at the level of individual cell types, which is particularly relevant for complex conditions such as autism spectrum disorder (ASD) [234].

Aberrant DNA methylation is also implicated in a range of non-neurological conditions, including fibrosis, autoimmune disease, and chronic inflammation. In fibrotic disorders, hypermethylation of anti-fibrotic genes contributes to the activation of pathogenic signaling pathways, whereas *DNMT* inhibitors have been shown to restore gene expression and reduce tissue damage in preclinical models [235]. In autoimmune diseases such as systemic lupus erythematosus (SLE) and Sjögren’s syndrome, hypomethylation of interferon-regulated genes appears to represent a shared pathogenic feature, and these methylation patterns can be used to distinguish disease states with relatively high accuracy [236]. Similarly, in rheumatoid arthritis, global hypomethylation affects genes involved in inflammation and cell migration, further supporting a role for epigenetic dysregulation in disease pathogenesis [237]. cfDNA methylation profiling can also be used to infer tissue of origin across multiple organ systems. Reference methylation atlases indicate that plasma cfDNA contains signals derived from specific tissues and can capture shifts associated with different disease states [122]. More recent work using deep learning-based deconvolution has improved this approach further, enabling more sensitive detection of tissue injury across a range of conditions, including cardiovascular, renal, and neurological diseases [238]. Across these diverse disease settings, several common patterns become apparent. DNA methylation changes act not only as disease-specific regulatory mechanisms, but also as broader contributors to cellular plasticity, immune modulation, and tissue dysfunction.

**Figure 5:**
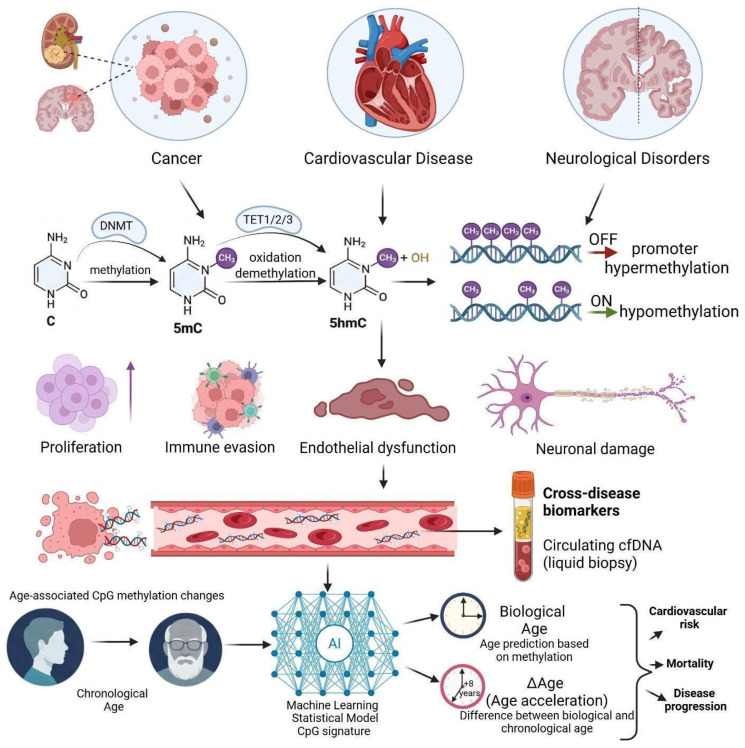
DNA Methylomics Across Human Disease Domains: Cancer, Cardiovascular, and Neurological Disorders. Schematic overview of DNA methylation dysregulation across major disease domains, highlighting both disease-specific alterations and shared regulatory mechanisms. The figure emphasizes the convergent translational potential of cfDNA methylation profiling as a cross-disease, non-invasive biomarker platform for diagnosis, monitoring, and tissue-of-origin inference.

One important implication is that these signals can be detected in circulating cell-free DNA. This creates a shared translational framework that extends beyond oncology and opens the possibility of cross-disease, non-invasive monitoring of tissue damage and disease progression (Figure 5).

## 7. Future Perspectives and Conclusion

The integration of high-resolution methylomic profiling with artificial intelligence and computational pathology is gradually moving the field beyond descriptive epigenomics toward more predictive and clinically actionable frameworks. This shift is already visible in several areas. For instance, DNA methylation-based classification of central nervous system tumors has reshaped diagnostic categories and is now incorporated into WHO guidelines [239]. A comparable trend is emerging in rare genetic disorders, where genome-wide methylation episignature analysis has proven useful in resolving cases that remain unexplained after conventional genetic testing [240]. Taken together, these developments suggest that the main barriers to clinical implementation are no longer primarily technological. Instead, they increasingly relate to standardization, regulatory alignment, and the demonstration of clinical and economic value [241]. Despite this progress, important challenges remain. Differences between analytical platforms and the lack of standardized workflows continue to affect reproducibility across studies [242]. In parallel, the under-representation of diverse populations in reference datasets raises concerns about generalizability and equity. Because DNA methylation patterns are influenced by both genetic ancestry and environmental exposures, future studies will need to account for this variability more systematically. Addressing this issue will be essential for ensuring that methylation-based tools can be applied reliably across populations [243]. The development of comprehensive, open-access reference methylome atlases represents a logical next step. Integrating methylomic, transcriptomic, and proteomic data at high resolution will make it possible to move toward a more systems-level understanding of disease. Foundational initiatives, including the human cell-type methylation atlas and the NIH Roadmap Epigenomics Consortium, have already established a strong basis for this effort [134,244]. Expanding these resources to include diverse populations and a broader range of disease states will be critical for advancing precision methylomic medicine. Ultimately, further progress will depend on how effectively multi-omic integration, spatial and single-cell technologies, and machine learning can be combined. These approaches are expected to enable the construction of more detailed and functionally annotated epigenomic maps of human disease. Achieving this will require coordinated international efforts, greater standardization of analytical pipelines, and sustained attention to population diversity. Together, these factors will determine whether advances in methylomics translate into clinically meaningful and broadly accessible precision medicine.

## Figures and Tables

**Table 1:** Overview of publicly available databases.

Resource	Omics-Types	Epigenomic Coverage	Scope	Key Feature
TCGA	Genomics, Epigenomics, Transcriptomics, Proteomics	HM27/HM450/EPIC beta-values	33 cancer types, >20,000 samples	Pan-cancer reference standard
GEO	Epigenomics, Transcriptomics, Genomics	DNA methylation (WGBS, Illumina arrays, methyl-seq), ChIP-seq (histone marks), ATAC-seq (open chromatin)	>200,000 studies, 6.5 million samples	Cross-disease multi-species community archive;
Ensembl/ ENCODE/ Roadmap	Epigenomics, Transcriptomics	Histone modifications, TF ChIP-seq, ATAC-seq, DNase-seq, DNA methylation,	123 epigenomes (Ensembl 95); 127 reference epigenomes	Integrates ENCODE + Roadmap + Blueprint data
FANTOM	Genomics, Epigenomics, Transcriptomics	ChIP-seq, DNase-seq, ATAC-seq, Bisulfite-seq	Mammalian genomes (human + mouse focus); >1000 profiles by CAGE in FANTOM5	Integrates The International Human Epigenome Consortium (IHEC), ChIP-Atlas
scMMO-atlas	Genomics, Epigenomics, Transcriptomics, Proteomics	scATAC-seq (chromatin accessibility at single-cell resolution)	3,168,824 cells from 27 cell tissues/organs	Joint scATAC + RNA identifies new cell subsets; includes disease samples (Alzheimer’s, COVID-19, brain tumors)
DriverDBv3	Genomics, Epigenomics, Transcriptomics	~12,000 methylation datasets; methylation driver identification (joint methylation + expression analysis); DNA methylation–expression correlation;	Pan-cancer, ~3000 RNA-seq, ~2000 exome-seq, ~11,000 CNV, ~12,000 methylation datasets	Synergistic gene-pair survival analysis (HR >1.5-fold threshold), methylation + CNV + miRNA driver identification
MLOmics	Genomics Epigenomics Transcriptomics	DNA methylation beta-values at promoter level (500 bp upstream + 50 bp downstream of TSS), region-level beta-values, median-centering normalization via limma, lowest-methylation promoter selection for multi-promoter genes	8314 patient samples, 32 cancer types, TCGA source, 20 ML tasks	ML-ready with 3 feature versions (Original, Aligned, Top-ANOVA), pre-built 6–10 baseline models per task, bio-knowledge linking

**Table 2:** Comparative Performance of ONT Duplex and PacBio HiFi CCS for Clinical Methylation Detection.

Parameter	ONT Duplex (R10.4.1HD)	PacBio HiFi CCS
Detection principle	Ionic current disruption of native DNA through protein nanopore	Polymerase kinetics during SMRT sequencing of native DNA
Read accuracy	Q30 consensus; error rate < 10^−7^ (duplex) [81,82]	>99.91% precision/recall for SNVs and indels [85]
Typical read length	Tens to hundreds of kilobases	15–25 kb (HiFi mode)
Modifications detected	5mC, 5hmC, 6mA simultaneously [62,63]	5mC (primary); 5hmC (emerging support)
Key software	Dorado, Remora, DeepMod2, Uncalled4 [62,71,72]	ccsmeth (BiGRU), modbamtools, ccsmethphase [64,65]
5mC sensitivity	>90% (R10.4 chemistry) [67,69]	>90%; high correlation with bisulfite-seq [64,65]
Haplotype phasing	MethPhaser; N50 extended 78–151% [76]	ccsmethphase; allele-specific methylation [64]
cfDNA applicability	Limited—short circulating fragment [94]	Limited—same constraint applies [94]
Clinical advantage	Portable; rapid turnaround; real-time calling	Superior indel accuracy; imprinting disorder gold standard
Key limitation	Higher raw error in simplex; duplex yield ~30–50%	Lower throughput; higher reagent cost per base

## Data Availability

All research data are available in this manuscript.
